# Multiple behavioural, morphological and cognitive developmental changes arise from a single alteration to early life spatial environment, resulting in fitness consequences for released pheasants

**DOI:** 10.1098/rsos.160008

**Published:** 2016-03-23

**Authors:** Mark A. Whiteside, Rufus Sage, Joah R. Madden

**Affiliations:** 1 Centre for Research in Animal Behaviour, Psychology, University of Exeter, Exeter EX4 4GQ, UK; 2 Game and Wildlife Conservation Trust, Burgate Manor, Fordingbridge, Hampshire SP6 1EF, UK

**Keywords:** anti-predator behaviour, development, habitat complexity, roosting, spatial cognition, survival

## Abstract

Subtle variations in early rearing environment influence morphological, cognitive and behavioural processes that together impact on adult fitness. We manipulated habitat complexity experienced by young pheasants (*Phasianus colchicus*) in their first seven weeks, adding a third accessible dimension by placing elevated perches in their rearing pens mimicking natural variation in habitat complexity. This simple manipulation provoked an interrelated suite of morphological, cognitive and behavioural changes, culminating in decreased wild mortality of birds from complex habitats compared with controls. Three mechanisms contribute to this: Pheasants reared with perches had a morphology which could enable them to fly to the higher branches and cope with prolonged roosting. They had a higher propensity to roost off the ground at night in the wild. More generally, these birds had more accurate spatial memory. Consequently, birds were at a reduced risk of terrestrial predation. The fitness consequences of variation in early rearing on behavioural development are rarely studied in the wild but we show that this is necessary because the effects can be broad ranging and not simple, depending on a complex interplay of behavioural, cognitive and morphological elements, even when effects that the treatments provoke are relatively short term and plastic.

## 1. Introduction

The development of an individual's morphology, behaviour and cognitive performance can be highly plastic, shaped by conditions experienced early in life [1–3]. Consequently, the fitness in terms of the survival or reproductive success of individuals is strongly influenced by early life environment either in concert with, or independent of, their genotype [4,5]. One facet of early life influencing development across a range of taxa is the spatial complexity of the habitat experienced during rearing. Increased habitat complexity may alter development in three, non-exclusive ways. First, a complex habitat may permit individuals to escape attacks by others, by hiding or fleeing behind barriers. This can reduce the levels of stress experienced [6], or reduce the value of investing in a dominant phenotype (e.g. [7]). Second, a more complex environment can initiate morphological changes that mean the individual can move more effectively through such complex habitats [8–10]. Finally, a complex habitat may provide enhanced opportunities for learning, which in turn affects neural development, cognitive performance and adult behaviour [11–13], producing differential performance in spatial cognition tasks [14] or speed in finding shelters [6]. These processes are unlikely to be independent of one another, but rather form part of a suite of coordinated changes; for example, reduced exposure to early life stressors mediates neural and cognitive development [15]. However, few studies take a holistic approach to consider the range and interplay of effects that a single early life perturbation may induce, and fewer still combine controlled early life conditions with fitness consequences for adults in the wild.

Pheasants (*Phasianus colchicus*) naturally nest in a variety of micro-habitats ranging from open grassland to established woodland both in their native range in east and central Asia, and in areas where introduced [16,17], meaning that the spatial complexity of the rearing micro-habitat will depend on features of the local environment. In artificial situations, pheasants and other gamebirds are typically reared in unnatural and often spatially simple habitats before release into the wild for recreational hunting [16,18]. We concentrated on the spatial environment that chicks experienced early in life, and asked specifically how access to elevated perches may provoke complex and long-lasting behavioural, morphological and cognitive developments that impact the fate of released birds. High mortality of reared galliformes is observed in the first weeks after release into the wild when the main cause is predation by foxes (*Vulpes vulpes*), terrestrial, crepuscular predators [19–21]. Birds naturally use elevated perches to roost at night as a form of anti-predation behaviour [22,23], and in the wild, pheasant mothers give calls which draw chicks up to roost on elevated perches [24]. Poor roosting behaviour is responsible for high mortality in released cheer pheasants (*Catreus wallichi*) [25] and grey partridge (*Perdix perdix*) [26]. An absence of perches during early development may inhibit individual opportunities for learning and morphological development necessary for this essential behaviour. Two mechanisms related to early life access to perches may influence chances of survival upon release; (i) specifically, the development of a functional anti-predator behaviour, in this case roosting, including the propensity to roost and the morphological characteristics required for this, and (ii) more generally, the cognitive and social influences driven by a more spatially complex environment, affecting brain development and cognitive ability, which have fitness benefits beyond simple roosting behaviour. Therefore, we predict that individuals reared in a spatially complex environment with access to perches will exhibit differential morphological, behavioural and cognitive development trajectories which reduce their mortality as adults in the wild.

## 2. Material and methods

### 2.1. Rearing and release into the wild

We reared and released pheasant chicks in 2013 on the Middleton Estate, Hampshire. The estate hosts a game shoot from October until February and employs gamekeepers to manage the release of pheasants through habitat management, providing supplementary food and controlling predator numbers. In May, we purchased 900 one-day-old pheasant chicks from a commercial supplier. Chicks were marked using individual numbered plastic patagial wing tags (18 × 4 mm Roxan Ltd) and randomly allocated to three treatment groups, differing in their access to perches. At three weeks, all birds were fitted with a larger wing tag (30 × 80 mm) with numbers matching those of the small tag. These large tags could be read by observers in the field at approximately 50 m. In treatment 1, a control, chicks were reared under standard commercial rearing conditions with no access to perches. In treatment 2, chicks were reared under the same standard rearing conditions as the control treatment, but with access to natural perches, in the form of approximately 20 mm diameter hazel (*Corylus avellana*) boughs. In treatment 3, chicks were reared under standard rearing conditions with access to artificial perches, in the form of 20 mm diameter plastic conduit piping.

We established 10 replicates of each treatment, with each replicate containing 30 chicks, housed separately in a heated shed (1.3 × 1.3 m) for the first two weeks of life. For the next five weeks, each replicate had access to a separate open grass run (1.3 × 6.8 m), as well as their shed. For treatments 2 and 3, there were three perches inside the shed (total length = 2.8 m) and a further three perches (total length = 3.6 m) in the open grass run. The current minimum welfare recommendations for intensively reared adult chickens suggests a perching distance of 0.15 m per bird [27,28], we provided a total of 0.21 m per bird and therefore confident that the chicks had enough room for all to perch.

Water and age-appropriate commercial game feed was available ad libitum throughout the rearing period. To maintain stocking density, any bird that died during the rearing period was replaced. Replacement chicks were reared in conditions analogous to the control and excluded from subsequent analyses. A preliminary analysis of the data in all subsequent questions revealed no differences between the groups reared with artificial or natural perches. Therefore, we combined birds from the two treatments for the remainder of our analyses and simply compared those reared with perches against those without. The birds were simultaneously being subjected to a dietary manipulation [29] in a fully balanced 3 × 2 design, where birds were either fed a control or an enriched diet including mixed bird seed and live mealworms. Our preliminary analyses revealed no significant effects of diet on measures being considered in this paper, so we either combined data from both diets when conducting non-parametric analyses, or included diet as a factor in our models.

In July, at seven weeks old, the birds from all treatments were mixed together and placed into one of two release pens on the estate. The pens each measured approximately 12 000 m^2^ and contained woodland, open areas of grass and dense patches of understorey as well as supplementary food and water. All pens were surrounded by wire and electric fences to exclude terrestrial predators, but were unroofed, so birds were exposed to the threat of avian predation. Birds could leave and re-enter the pen at will.

### 2.2. Measuring mortality in the wild

Mortality in the wild was measured using two methods. Prior to the start of the breeding season in April, birds that died of natural causes were collected by searching the estate and surrounding areas. The extent that we could search the area depended on the season. Post-release and prior to the hunting season (June–October), we conducted daily searches of areas of the estate. During the hunting season (October–February), the area was visited less frequently but more methodically as beaters, engaged in driving the game towards the waiting guns, were informed of the project and searched for carcasses and tags as they walked through the estate. After the shooting season (March–June), the same area was visited about once a week. In the breeding season, we conducted a more detailed analysis of a subset of 20 females (14 reared with perches, six reared without perches) and six males (four reared with perches, two reared without perches) which survived the winter and shooting season and were caught using baited, walk-in traps set at feeder sites. We captured birds in March using walk-in traps set around feeder sites and attached radio tags equipped with mortality switches to pheasants from known rearing conditions. We followed the fates of these birds over the following four months, radio-tracking birds around twice a week, access permitting. For both the initially released and radio-tagged birds, we picked up carcasses of the pheasants that we found and identified them by their numbered wing tag. Some dead birds were damaged, indicating that they had been predated. If there was no external damage to the bird, we suspect that it died of other causes, perhaps disease or starvation. However, it is possible that birds we recovered with marks of predation had actually died of other causes and their body had been scavenged. Therefore, we could not confidently discriminate natural causes of death so we combined them into a single category of natural deaths. We tested whether the number of birds dying in the first year after release differed between treatments. We also used a third, proxy, measure of natural survival, considering the numbers of birds from each treatment that were shot during the hunting season. Interpreting data collected from shot birds can be problematic due to unexpected biases of age [30], sex [31] or personality [32]. We used it simply as confirmation that there were more treatment birds alive than control birds at the start of the shooting season, rather than unsupported evidence in its own right that our treatment affected mortality early in life. If more control birds than treatment birds had been shot, then we would have been suspicious that our methods of searching for and finding carcasses that died of natural effects were unreliable.

### 2.3. Measuring dispersal from the release site

Neighbouring estates and shoots were informed of the study and were asked to provide us with details of birds that they shot. We used a binomial test to ask whether birds shot off the release site differed across rearing treatments.

### 2.4. Measuring levels of early life aggression

We collected 10 min focal follows from individually identified birds from all houses, for all seven weeks of the study (27–30 of each sex per week for the first six weeks; 19 of each sex for the last week). Each bird was observed once. We measured the number of agonistic interactions which included aggressive pecks, fights and chases directed towards or received by the focal individual. We used a general linear mixed model (GLMM) to ask whether the combined number of agonistic interactions per hour differed with rearing treatment, sex and age as variables, with house as a random factor. Initially, the full model with all likely explanatory variables was entered into the model including all possible two-way interactions. Terms were then sequentially dropped until the minimum adequate model (lowest Akaike's Information Criterion) contained only variables whose elimination would reduce the explanatory power of the model.

### 2.5. Measuring immediate effects of early access to perches on morphology

We recorded the mass (Slater Super Samson spring balance—precision 5 g) and tarsus thickness, described as the width at the thinnest part of the tarsometatarsus directly below the spur (e.g. [33]), of all birds upon release into the wild at seven weeks old. A GLMM was used to ask whether morphometrics differed with early access to perches, considering sex differences, with the rearing house as a random factor.

### 2.6. Measuring spatial memory: eight arm radial maze test

We presented 27 six-week-old chicks, nine randomly chosen from each treatment, with a test of their spatial working memory. We used a well-established and widely deployed test paradigm (e.g. [34]); an eight arm radial arm maze [35]. In the centre of the radial arm maze was a circular starting arena and at the end of each arm (0.8 m long) was a reward hidden by a barrier. The chicks became accustomed to the arena for 20 min a day, twice a day for 4 days. On days 1 and 2, the birds were exposed to the radial maze in groups of three individuals with rewarded food (chick crumb and dead mealworms). On day 3, the birds were exposed to the radial maze singly with rewarded food. On day 4, the birds were exposed to the radial maze singly with the rewarded food placed only behind the goal. Testing started after the fourth day of training. To solve the test correctly, a bird entered each arm once and only once and ate the reward (a live mealworm). If a bird re-entered an arm (by at least one half of its body length) where they had already eaten the mealworm it was recorded as an error. We recorded the number of errors and stopped the test after 15 min if not all eight arms had been visited. The arena roof was covered in fine mesh which enabled birds to look up to orientate, assisted by a large red feature (47 kg gas bottle) and the walls of the test room being different colours. The testing took place over 3 days between 07.30–10.00 and 18.30–20.00, to replicate natural foraging times and reduce the stress of moving and testing birds during the midday heat. Individuals deemed to have better spatial memory are those which make the fewest errors. A faultless individual would solve the maze within eight choices. All birds tested made eight or more choices. We used a general linear model (GLM) to ask whether the errors made during the first eight choices differed with early access to perches.

### 2.7. Measuring roosting behaviour in the wild

For the first two weeks after release into the wild, we determined how many birds from each treatment could be seen roosting on elevated perches at night. Between the 45 min before and 60 min after last light, we observed and individually identified birds in the release pens, using an IR illuminated night-vision monocular where necessary, to record which birds were roosting on perches off the ground. We conducted *χ*^2^-analysis to ask whether the number of birds seen roosting at night differed with early access to perches. We repeated these observations after the birds had lived for six weeks in the wild to determine whether there was a long-term effect of early access to perches on an individual's propensity to perch.

### 2.8. Measuring prolonged effects of early access to perches on morphology

To determine whether there was a long-term effect of rearing treatment on morphology, we collected all birds shot during the shooting season (*n* = 202) and weighed them and measured tarsus thickness within 4 hours of death. We also removed left tibias from 40 birds shot during the shooting season. They were stripped of soft tissue by hand and the bone cap removed. The bone length was measured using a calliper and bones were weighed on a precision balance. A midline was marked and the maximum and minimum outer diameter (diaphysis diameter) was measured. Using a saw, we cut through the midline and measured the maximum and minimum medullary canal diameter. Three indices were then calculated based on the following formulae: (i) tibiotarsal index = [(diaphysis diameter − medullary canal diameter)/diaphysis diameter] × 100 [36]; (ii) robusticity index: bone length/cube root of bone weight [37] and (iii) bone weight length index: bone weight/length [38]. A general linear model was used to test whether early access to perches affected the different morphometrics, while controlling for sex differences. We initially used a GLMM including rearing house as a random factor, but it had no effect, probably because several months had elapsed since birds had left the houses, so we dropped it and simply ran GLMs.

## 3. Results

### 3.1. Effects of early access to perches on mortality in the wild and dispersal from the release site

Birds reared with perches were less likely to be found dead of natural causes immediately post-release and through their first winter. Fourteen of the 296 birds (5%) that had been reared under the control conditions were detected as having died of natural causes after release prior to the start of the breeding season compared with only four of the 585 birds (less than 1%) that had been reared with perches (binomial test: *p* = 0.002). A higher proportion of birds that had been reared with perches (150 of 585 released, 26%) were shot compared with those reared under control conditions (65 of 296 released, 22%) (binomial test: *p* = 0.14). Early access to perches did not affect the number of birds dispersing from the release site and being shot on neighbouring shoots (birds reared with perches = 15, without perches = 12: binomial test: *p* = 0.17).

This effect of early rearing environment was lost once the shooting season ended and the breeding season began. Of the 18 birds reared with perches who we radio-tagged in March, 13 were dead of natural causes by the end of the breeding season in July, compared with three of the eight birds that had been reared without perches (*χ*^2^ = 1.55, *p* = 0.21).

### 3.2. Immediate effect of early life access to perches

#### 3.2.1. Aggression

**Figure 1.**
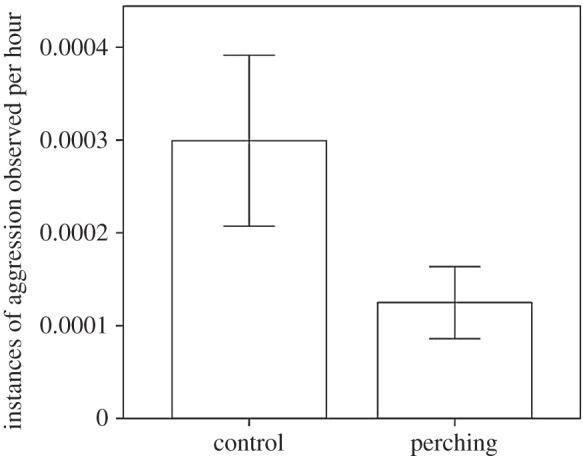
The average combined instances of aggression per hour for birds reared with and without access to perches. Error bars indicate ±1 s.e.

Birds reared without access to perches were subjected to higher combined levels of aggression per hour than birds reared with perches (GLMM: Perches: *F*_1,269_ = 4.24, *p* = 0.041; figure 1).

#### 3.2.2. Morphology

**Figure 2.**
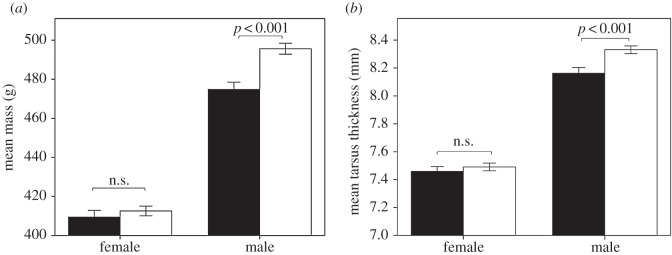
The mass (*a*) and tarsus thickness (*b*) of birds reared with access (white bars) and without access (black bars) to perches at seven weeks old when they were released into the wild. Error bars indicate ±1 s.e.

Early access to perches affected the mass of a bird upon release (GLMM: Perches: *F*_1,874_ = 13.79, *p* < 0.001), which was marked in males, with males reared with access to perches being heavier than males reared without access to perches (*post hoc* test *p* < 0.001) (GLMM: Perches × Sex: *F*_1,874_ = 7.59, *p* = 0.006; figure 2*a*). As expected, males were heavier than females (GLMM: Sex: *F*_1,874_ = 527.36, *p* < 0.001). Early access to perches also affected the tarsus thickness of birds at the time of release (GLMM: Perches: *F*_1,879_ = 8.83, *p* = 0.003; figure 2*b*), which was primarily driven by tarsus thickness differences in males (*post hoc* test *p* < 0001; GLMM: Perches × Sex: *F*_1,876_ = 4.15, *p* = 0.042). As expected, males had thicker tarsi than females (GLMM: Sex: *F*_1,879_ = 530.70, *p* < 0.001).

#### 3.2.3. Spatial cognition

**Figure 3.**
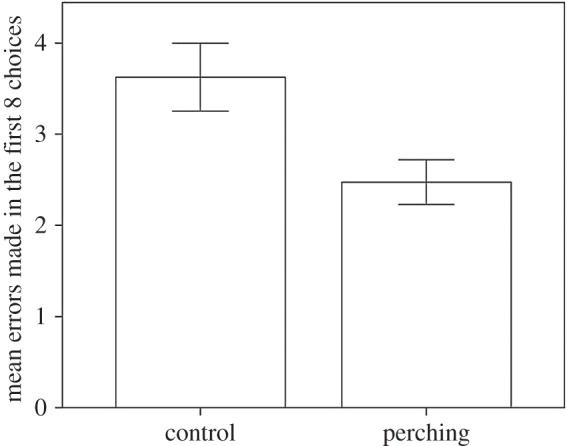
The mean number of errors made for the first eight choices when presented with an eight arm radial maze for birds reared with and without access to perches. Error bars indicate ±1 s.e.

Birds reared without access to perches made more errors in their first eight choices than birds reared with access to perches (GLM: Perches: *F*_1,23_ = 6.07, *p* = 0.02; figure 3). The effect of treatment was not affected by the sex of the bird (GLM: Perches × Sex: *F*_1,23_ = 1.75, *p* = 0.20). Control birds made approximately four mistakes, behaving as if they were visiting arms purely at random (1-sample *t*-test *x* = 4, *t*_7_ = 1.00, *p* = 0.35). Treatment birds made approximately two mistakes, and this differed from random visits (1-sample *t*-test *x* = 4, *t*_18_ = −6.20, *p* < 0.001) indicating that they had indeed ‘learned’ the problem.

### 3.3. Post-release effects of early life access to perches

#### 3.3.1. Morphology

There was no prolonged effect of early access to perches on the mass of birds recorded when they were shot several months after release (GLM: Perches: *F*_1,118_ = 0.97, *p* = 0.33), and males did not show the same difference in mass between treatment groups as previously shown at release (GLM: Perches × Sex: *F*_1,118_ = 0.37, *p* = 0.55). Likewise, differences in tarsal size were lost (GLM: Perches: *F*_1,163_ = 0.67, *p* = 0.42). There was also no difference in bone mineralization in these shot adult birds (tibiotarsal index GLM: Perches: *F*_1,29_ = 2.49, *p* = 0.13; robusticity index GLM: Perches: *F*_1,29_ = 2.03, *p* = 0.17; bone weight/length index GLM: Perches: *F*_1,29_ = 2.54, *p* = 0.12).

#### 3.3.2. Natural roosting behaviour

**Figure 4.**
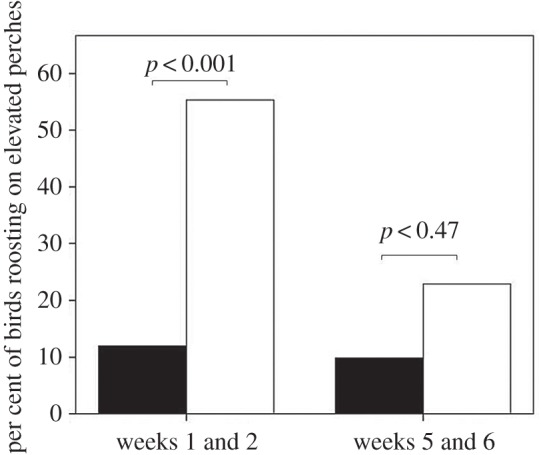
The percentage of birds observed roosting on elevated perches from birds reared with access (white bars) and without access (black bars) to perches during the first two weeks after release and in weeks five and six after release.

More birds reared with access to perches were observed roosting at elevation at night in the wild during the first two weeks after release than birds reared with control conditions ($\chi_{c}^{2}=15.82,\;$ *p* < 0.001; figure 4). This difference across the treatments disappeared after six to seven weeks, with no more perch-reared birds roosting at elevation than control birds ($\chi_{c}^{2}=0.53,\;$ *p* = 0.47; figure 4).

## 4. Discussion

A simple difference in the environment experienced in the first seven weeks of life by pheasants, specifically the opportunity to perch off the ground, provoked a coordinated suite of short-term and reversible changes to individual morphology, cognitive performance and behaviour which corresponded to differences in mortality during a critical stage of life. Males raised in the more spatially complex habitat were heavier, had thicker tarsi, exhibited more accurate memory of locations in a radial maze task and were subject to lower levels of aggression, prior to release.

The initial enhancement of body mass and skeleton is probably due to the spatial complexity of their rearing environment, because our experimental and analytical design controlled for alternative explanations such as diet. Complex habitats provide an incentive to fly to perches [8]. In addition, small diameter perches, such as we used, induce birds to a wing-flap to balance [39]. Both these features help develop the associated pectoral and thigh muscles, and this may bring benefits beyond improved roosting ability. Increased pectoral muscle mass increases take-off power which benefits predator evasion [40]. Poor development of flight muscles due to lack of perches during development may explain the poorer flying ability of captive-reared birds compared with wild ones in terms of shorter flight distances [41,42], endurance [43] and take-off ability [44]. This reduced early life flight performance may have fitness consequences through reduced ability to escape terrestrial predation, independent of the use of roosts. Improved flying ability may lead to bias in sampling based on shooting, with better flyers being faster and thus harder to kill. This may explain why, although we recorded a higher proportion of birds raised with perches being shot compared with control birds, the difference was not as marked as that we recorded based on birds killed by natural causes.

A second broader consequence of early exposure to elevated perches is enhanced spatial ability, revealed in performance in the radial maze task. Chickens with access to perches are better at using three-dimensional space [14], and rearing without perches can impair spatial cognitive tasks, such as navigating the environment [45]. Complex habitats may cause gross neural changes as well as more subtle behavioural modifications. Increased habitat complexity in early life led to larger brain regions in salmonids [11]. An enhanced spatial ability may bring additional fitness benefits beyond simply a propensity to perch. It may permit better memory of food or shelter sites [46], more direct movement between sites [47] or increased object exploration [48]. Such effects may persist even when the initial trigger for differential development, that is access to perches, has been removed.

A third effect was reduced levels of aggression. Access to perches may offer an opportunity to escape aggression from dominant individuals, with subordinates using the raised positions. Spatially complex environments also make individuals less likely to adopt a potentially costly aggressive phenotype [49]. Reduced exposure to aggression may permit individuals to exhibit higher feeding rates [50], and engage in less risky behaviour as adults [51]. Young pheasants interact violently as they assert dominance, and this can be marked in confined, unenriched commercial rearing systems [52]. Exposure to early life stressors, such as aggression, influences behavioural, morphological and cognitive development across a wide range of taxa [3], and frequently does so by modulating particular regions of the brain [53]. A more spatially complex environment early in life allows young to escape stressful aggression, and this in turn may mediate differential neural development in brain regions related to spatial ability.

These differences in morphology, cognitive performance and behaviour were accompanied by differences in roosting behaviour upon release. Birds raised with access to perches were more likely to roost off the ground at night immediately after release into the wild. Similar results were observed in captive galliformes, where chickens reared with perches in captivity were shown to perch more readily as adults [45,54], in particular at night [55].

We suspect that this nocturnal roosting explained the decreased mortality of birds reared with perches. Roosting at night is an essential anti-predation behaviour for ground-dwelling birds [22], especially where the crepuscular and terrestrial fox is the biggest cause of predation [21,56]. A failure to roost on elevated perches may explain the extremely high mortality (up to 65% in the first week [57] or 81% in the first month [19]) experienced by released pheasants reared in conventional, unenriched conditions.

Our observed levels of mortality (5% for control birds compared with 1% for treatment birds) were markedly lower than those reported by other studies. This is because previous studies have typically radio-collared a relatively small sample (e.g. 74 pheasants [19]) and then followed the fates of all individuals. By contrast, we tagged a much larger sample but could retrieve only a small sample of these birds through unguided searching. Carcasses equipped with radio collars can be detected even when buried in underground caches, or hidden in dense undergrowth. We could not afford to radio-collar such a large sample, nor could we rigorously search more than 4000 acres. Therefore, our mortality measures should not be directly compared with other published figures based on very different tagging and search methodologies. Instead, our unbiased retrieval methods provide a relative comparison of mortality between the rearing treatments. Dispersal from the release site did not differ with rearing treatment. Consequently, we do not believe that our finding more control birds dead of natural causes was because those reared with perches had simply dispersed from the study area.

In our study, the lower mortality of birds raised with perches may be exaggerated by frequency dependency: both birds reared with and without early access to perches were released at the same site and predators may have preferentially taken the easier prey not using elevated perches in order to satisfy their hunger. If all the birds had been raised with perches, predators may still have eaten as many birds in total, but simply had to work harder to obtain them, perhaps by switching to more diurnal hunting patterns.

Lower mortality corresponding to early life environment was only observed for the first few months post-release, with the effects being lost by the end of winter and over the breeding season. The loss of differences in mortality later in the year may have two explanations. First, the selective pressures faced by birds in the breeding season, especially females who comprised the majority of our tracked birds, are likely quite different from those faced by newly released birds. Wild hens usually nest on the ground and thus elevated roosting provides them with no protection from terrestrial predators during the breeding season [16]. In addition, supplementary food usually provided by gamekeepers is typically withdrawn at the end of the shooting season, mimicking a seasonal food shortage over winter so birds of both sexes face starvation and this becomes the primary driver of mortality in winter and early spring [58], whereas recently hatched or released birds are surrounded by plentiful natural insects, fruits or grains, or supplementary feed hoppers from August until February, such that during this period, predation is the primary cause of mortality. Second, the loss of difference in mortality was matched by a corresponding loss of morphological (mass and tarsus thickness) and behavioural (elevated roosting) differences suggesting that birds from the control rearing condition who survive the first few weeks post-release may rapidly compensate for their early life deficiencies, learning, either individually or socially, to move and navigate in a third dimension and so develop an appropriate morphology. Mother pheasants can promote climbing and flying up to roost with the use of calls [24], and being a gregarious species, pheasants may follow peers who were raised with elevated perches as they ascend to roosts in the evening. Alternatively, it may simply be that direct access to perches facilitates individual learning of roosting behaviour. This change in behaviour was matched by compensatory morphological changes. Although mass and tarsal thickness was greater in seven-week-old birds raised in complex environments prior to release, matching effects seen in chickens reared in captivity with access to perches [10,59,60], these differences were lost over subsequent months. The mass and tarsal size and strengths of birds that had been shot at least three months later were not distinguishable by rearing environment. However, this behavioural and morphological compensation may not be rapid enough to equalize mortality risk. It is during this first month post-release that predation of pheasants is highest [19,57], explaining our observed differences in mortality.

Our manipulation of a single aspect of early life environment, namely habitat complexity, clearly illustrates the broad and interwoven range of proximate and ultimate consequences arising from this simple change. Many studies of developmental plasticity, especially those concentrating on behaviour, concentrate on single effects or a small number of tightly interrelated effects and seldom relate them to tangible fitness consequences such as mortality or reproductive success. Buchanan *et al.* [3] reviewed how early life conditions affect behaviour by modifying cognitive performance. None of the 36 studies of non-human vertebrates they considered explicitly measured fitness consequences. Further, only four of the studies considered the behaviour outside the laboratory, and of these, only one [61] involved experimental manipulation of early rearing conditions. Our work indicates that the effects of altered conditions during early life are unlikely to occur in isolation, but instead comprise part of a larger correlated suite of changes, which may have unexpected consequences. For example, enhanced spatial ability may exclusively facilitate better use of perches, but it may additionally affect foraging success [62] or home range use [63]. All these alterations are likely to influence fitness outcomes, and hence the way that selection acts on plasticity. Furthermore, the need to tightly control early life conditions in studies means that manipulations are typically carried out under captive conditions. This, coupled with the difficulty of releasing laboratory-reared vertebrates into the wild, makes assessing the consequences of the manipulation under natural conditions difficult. Therefore, our understanding of the evolutionary basis of developmental plasticity is likely to be incomplete unless we consider both the diverse range of effects which changes in any one early life factor initiates, and the fitness consequences for an individual in its natural environment.

## Supplementary Material

Supporting Data

## Data accessibility

The datasets supporting this article have been uploaded as part of the electronic supplementary material.
